# Distinct and overlapping functions of YAP and TAZ in tooth development and periodontal homeostasis

**DOI:** 10.3389/fcell.2023.1281250

**Published:** 2024-01-08

**Authors:** Jing Ma, Haixia Fan, Haixia Geng

**Affiliations:** ^1^ Department of Oral Medicine, Weifang Medical University, Weifang, Shandong, China; ^2^ Department of Oral Medicine, Jining Medical University, Jining, Shandong, China; ^3^ Department of Orthodontics, Affiliated Hospital of Jining Medical University, Jining, Shandong, China

**Keywords:** orthodontic tooth movement, YAP, TAZ, tooth development, periodontal homeostasis, tissue remodeling

## Abstract

Orthodontic tooth movement (OTM) involves mechanical–biochemical signal transduction, which results in tissue remodeling of the tooth–periodontium complex and the movement of orthodontic teeth. The dynamic regulation of osteogenesis and osteoclastogenesis serves as the biological basis for remodeling of the periodontium, and more importantly, the prerequisite for establishing periodontal homeostasis. Yes-associated protein (YAP) and transcriptional coactivator with PDZ-binding motif (TAZ) are key effectors of the Hippo signaling pathway, which actively respond to mechanical stimuli during tooth movement. Specifically, they participate in translating mechanical into biochemical signals, thereby regulating periodontal homeostasis, periodontal remodeling, and tooth development. YAP and TAZ have widely been considered as key factors to prevent dental dysplasia, accelerate orthodontic tooth movement, and shorten treatment time. In this review, we summarize the functions of YAP and TAZ in regulating tooth development and periodontal remodeling, with the aim to gain a better understanding of their mechanisms of action and provide insights into maintaining proper tooth development and establishing a healthy periodontal and alveolar bone environment. Our findings offer novel perspectives and directions for targeted clinical treatments. Moreover, considering the similarities and differences in the development, structure, and physiology between YAP and TAZ, these molecules may exhibit functional variations in specific regulatory processes. Hence, we pay special attention to their distinct roles in specific regulatory functions to gain a comprehensive and profound understanding of their contributions.

## 1 Introduction

**FIGURE 1:**
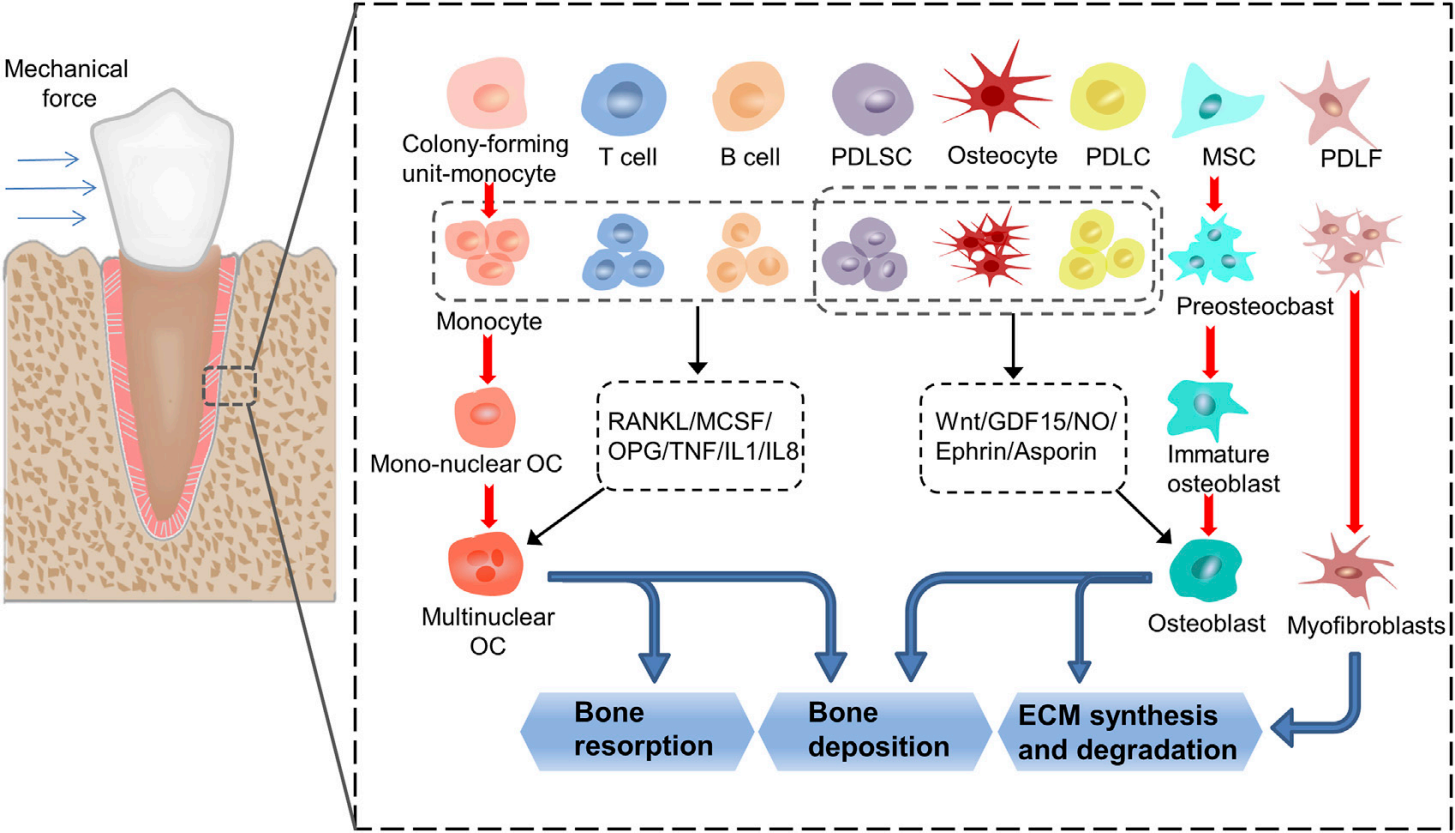
The process of transduction of mechanical into biochemical signals. When the teeth are subjected to force stimulation, the cells in the periodontal membrane and alveolar bone undergo a series of proliferation, differentiation, and apoptosis activities, and finally realize the remodeling of the periodontal tissue. In this process, monocytes, MSCs and PDLFs differentiate into osteoclasts, osteoblasts, and myofibroblasts, respectively. At the same time, T cells, B cells, PDLSCs, osteocytes and PDLCs affect the formation of osteoclasts by secreting cytokines such as RANKL, MCSF, OPG, TNF, IL1 and IL8, thereby regulate the resorption and deposition of bone tissue. PDLSCs, osteocytes and PDLCs affect the formation of osteoblasts by secreting cytokines such as Wnt, GDF15, NO, Ephrin and Asporin, thereby regulate the synthesis and degradation of ECM and the deposition of bone tissue. (PDLF: periodontal ligament fibroblasts; PDLSC: periodontal ligament stem cells; PDLC: periodontal ligament cells; MSC: mesenchymal cells; ECM: extracellular matrix).

Orthodontic tooth movement (OTM) involves the conversion of mechanical into biochemical signals within cells. The mechanical signals are transduced through extracellular matrix (ECM) to the mechanosensory cells, and the intracellular signaling pathways are activated. This leads to primary cell response, such as proliferation, differentiation, and apoptosis, ultimately resulting in tissue remodeling of the tooth–periodontium complex and OTM (Pavasant and Yongchaitrakul, 2011; Chukkapalli and Lele, 2018; Huang et al., 2018) (Figure 1). Periodontal remodeling and homeostasis are interconnected and serve as the foundation and prerequisite for each other during the development of teeth and periodontium. Li et al. proposed a new hypothesis on periodontal remodeling in OTM that builds upon the concept of “compression–tension” hypothesis. According to this hypothesis, periodontal ligament cells (PDLCs) and osteocytes are the primary sensors that respond to mechanical signals during OTM, where PDLCs control soft tissue remodeling, while osteocytes controls bone tissue remodeling, and the 2 cell types may interact with each other (Li et al., 2021).

Yes-associated protein (YAP) and transcriptional coactivator with PDZ-binding motif (TAZ) are downstream transcriptional coactivators of the Hippo signaling pathway. These molecules are involved in cell proliferation, apoptosis, and differentiation, and their influence extends to organ morphology, homeostasis, and tissue regeneration (Fu et al., 2017; Chen Y. A. et al., 2019; Moya and Halder, 2019). Deactivation of YAP and TAZ has been linked to impairments in tissue development, regeneration, and cell function, which contribute to the development of various diseases (Panciera et al., 2017). During tooth development, YAP or TAZ knockout can cause abnormal development of tooth germ and tooth morphology (Nagano et al., 2022; Pandya et al., 2022). In addition, recent studies have revealed that YAP and TAZ actively respond to mechanical stimuli and participate in the conversion of mechanical into biochemical signals, thereby regulating periodontal homeostasis and remodeling in OTM progress (Huelter-Hassler et al., 2017; Hulter-Hassler et al., 2017; Dasgupta and McCollum, 2019; Zarka et al., 2021; Nguyen and Lee, 2022). The inactivation of YAP and TAZ affects periodontal tissue remodeling and periodontal homeostasis, resulting in tissue structural disorders (Pandya et al., 2022). In contrast, the activation of YAP and TAZ has a significant effect on accelerating OTM and shortening treatment time. Therefore, the control of YAP and TAZ activity is crucial for tooth development and periodontal homeostasis. The spatiotemporal expression and localization of YAP and TAZ in the cytoplasm or nucleus play key roles in cell behavior and performing specific functions (Yang et al., 2014; Shiu et al., 2018; Cai et al., 2019; Kofler and Kapus, 2021). After mechanical stimulation, YAP and TAZ can migrate from the cytoplasm to the nucleus (Wan et al., 2023). In the cell nucleus, they interact with transcription factor TEA domain family members (TEADs) to control the expression of target genes, thereby affecting cell proliferation, differentiation, and apoptosis (Wan et al., 2023).

Notably, despite their similar functions, YAP and TAZ exhibit structural and physiological differences. There may be differences in how YAP and TAZ are regulated and how they interact with TEAD1–4. At the same time, YAP seems to have a greater effect than TAZ in terms of cell diffusion, control of cell volume, glucose uptake, proliferation, and migration (Plouffe et al., 2018). Therefore, YAP and TAZ may exhibit differences in the regulation of some functions while acting synergistically (Plouffe et al., 2018; Shreberk-Shaked et al., 2020; Jeong et al., 2021; Reggiani et al., 2021). At present, the effects of YAP and TAZ on tooth development and periodontal remodeling are incompletely understood and contradictory. Therefore, in this review, we summarize the roles of YAP and TAZ in tooth development and periodontal remodeling, with a focus on their unique contributions to the regulation of specific functions. The aim of this review is to provide a comprehensive and profound understanding of the mechanisms through which YAP and TAZ play their regulatory roles.

## 2 Differences in the structure and function of YAP and TAZ

**FIGURE 2:**
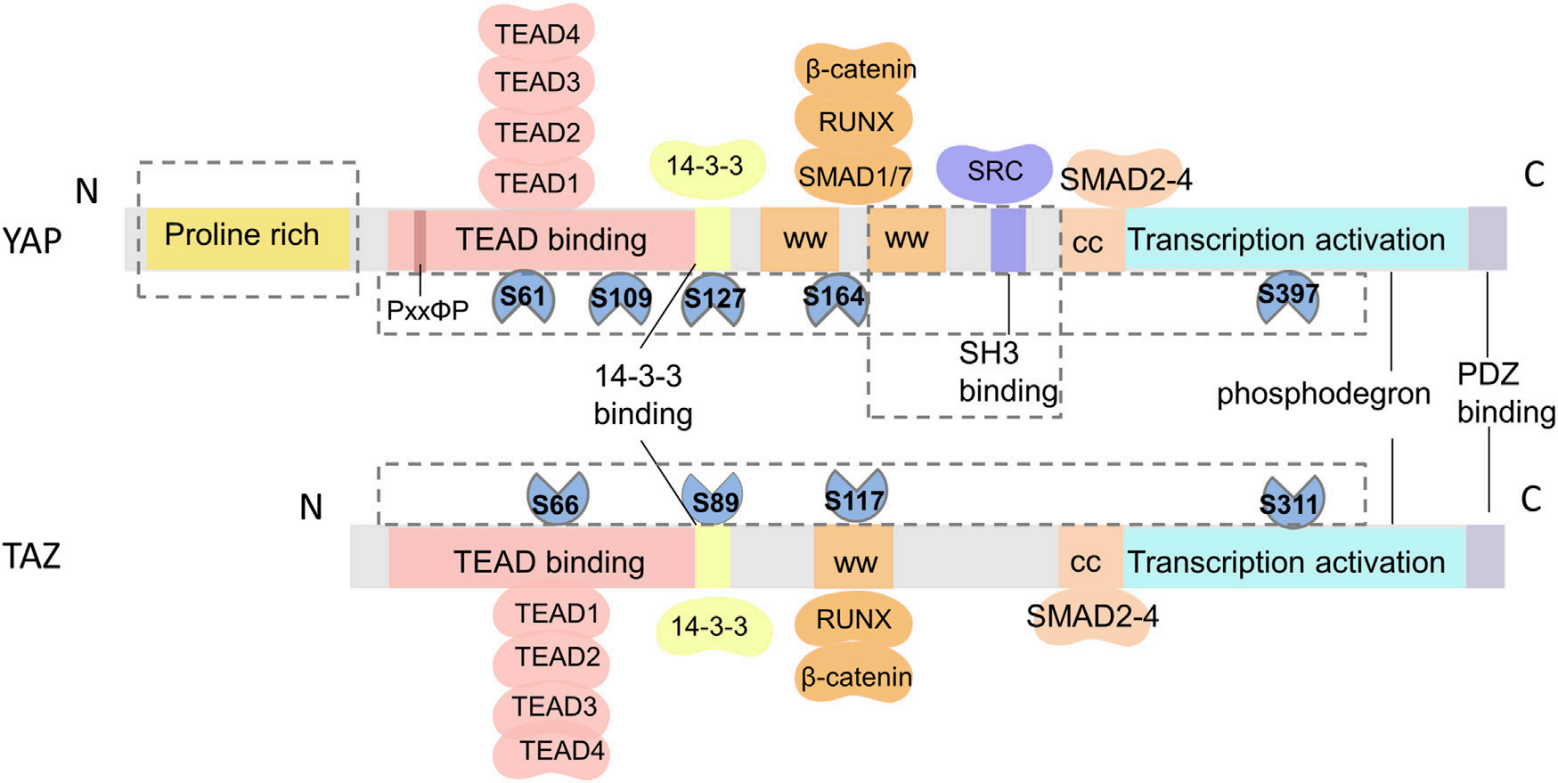
Schematic of the structural similarities and differences between YAP and TAZ. From N- to C-terminus, both YAP and TAZ have a TEAD-binding domain (TBD), WW domains (WW), coiled-coil region (CC), transactivation domain, and a PDZ-binding domain. TAZ only has one WW domain and lacks a proline-rich domain and an SH3-binding domain. The differences in phosphorylation sites and protein binding sites of YAP and TAZ are shown in the F.

The Hippo pathway has been highly conserved throughout evolution. YAP was first discovered in unicellular eukaryotes (Sebe-Pedros et al., 2012), while TAZ, a paralog of YAP, emerged much later in vertebrates (Hong et al., 2005). Structurally, YAP and TAZ have 45% similarity (Kanai et al., 2000), but there are notable distinctions between them (Figure 2). First, while both YAP and TAZ contain the WW domain capable of interacting with the PPXY and PDZ-binding motifs in the C-terminal region, YAP possesses two tandem WW domains, whereas TAZ has only one (Verma et al., 2018). Moreover, YAP includes an SH3-binding motif and an N-terminal proline-rich region involved in mRNA processing (Howell et al., 2004), which are not found in TAZ. Second, YAP and TAZ are regulated by different stability regulators, which control their activity and level in the nucleus. Finally, upstream serine/threonine kinases (LATS1/2) that phosphorylate YAP and TAZ are different. Phosphorylation at serine 127 (S127 and S89 in YAP and TAZ,respectively) promotes the translocation of YAP and TAZ to the cytoplasm, while phosphorylation at serine 397 (S397 and S311 in YAP and TAZ,respectively) leads to protein degradation (Zhao et al., 2010). Furthermore, the downstream TEAD1-4 domain, which interacts with YAP and TAZ, also exhibits dissimilarities (Li et al., 2010; Kaan et al., 2017). TAZ lacks the PxxΦP motif present in the N-terminal of YAP binding to the TEAD domain (Hau et al., 2013). Recent research has reported that TAZ-TEAD can form a heterotetrameric complex that may influence DNA target selection and induce the expression of specific target genes (Li et al., 2010). It has been suggested that these structural disparities between YAP and TAZ are responsible for their different functions *in vivo*.

In terms of functional regulation, YAP appears to exert a stronger influence than TAZ, and the inactivation of YAP has a pronounced effect on cell physiology (i.e., cell spreading, volume, granularity, glucose uptake, proliferation, and migration). YAP and TAZ also differ in terms of cell specificity, tissue specificity, protein stability and expression, and binding to transcription factors during development and regeneration (Plouffe et al., 2018). *In vivo* studies have demonstrated that YAP-knockout mice display embryonic lethality, defects in chorioallantoic attachment and yolk sac vascular development, and failure in embryonic axis elongation at E8.5 (Morin-Kensicki et al., 2006). Conversely, mice lacking TAZ develop polycystic kidney disease and emphysema (Makita et al., 2008; Lee et al., 2020). Moreover, YAP knockdown reduces the contractility of cancer-associated fibroblasts, leading to a decrease in collagen fiber production and elastic modulus, while TAZ knockdown shows minimal effects (Calvo et al., 2013). *In vitro* studies have demonstrated that both YAP and TAZ promote the proliferation of satellite cells. However, at later stages of myogenesis, TAZ enhances myogenic differentiation of myoblasts, whereas YAP inhibits such differentiation. During myogenesis, TAZ can regulate some genes independently of YAP (Sun et al., 2017). Therefore, YAP and TAZ play distinct roles in functional regulation through diverse mechanisms, but are often considered functionally redundant and have not yet been clearly differentiated.

## 3 YAP and TAZ regulate tooth development

**FIGURE 3:**
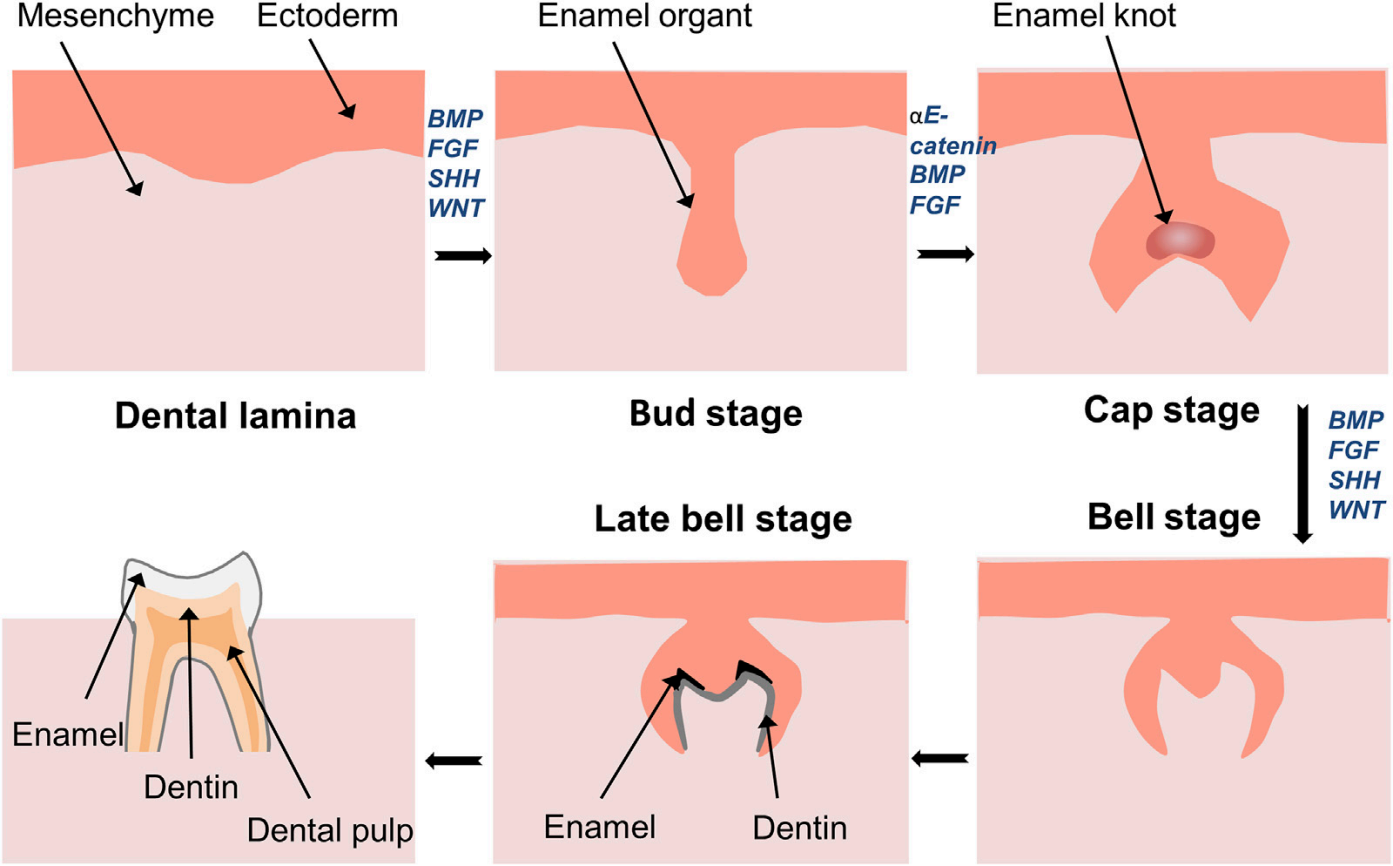
Development of tooth germ and formation of tooth tissue. Development of tooth starts from interaction of ectoderm and mesenchyme. Thickened dental lamina could form dental placode, and then it comes to the bud stage. Followed by the formation of enamel in the cap stage. The continuous growth of the cap stage will transfer into the bell stage. In the late bell stage, ameloblasts and odontoblasts differentiate to form enamel and dentin.

Tooth development is initiated through the interaction between the odontogenic epithelium and adjacent mesenchyme, which leads to the formation and differentiation of tooth germs, the development of tooth tissues, and eventually, the eruption of the tooth (Figure 3). This process relies on cell migration, proliferation, and differentiation. YAP and TAZ are continuously expressed from dental lamina formation to the whole development stage of tooth germ (Niki et al., 2021). A recent report has demonstrated that activation of Wnt/β-catenin signaling decreases the expression of YAP, leading to inactivation of YAP signaling, thereby resulting in abnormal tooth germ development (Nagano et al., 2022). The enamel knot acts as a signaling center and plays a vital role in guiding tooth morphogenesis and the formation of cusps (Thesleff et al., 2001; Yu and Klein, 2020). YAP is also involved in this process as a transcriptional activator that regulates cell proliferation (Kwon et al., 2015; Li et al., 2016). The overexpression of YAP in dental epithelial cells results in abnormalities in tooth morphology, such as widened dental lamina, mislocation of the enamel knot to the upper portion of enamel organ (ectopic cusp), and arrested tooth crown development (Liu et al., 2014; Kwon et al., 2015). In contrast, YAP-knockout mice exhibit small tooth germs with reduced epithelial cell proliferation (Liu et al., 2015). These findings suggest that YAP/TAZ may affect cell proliferation during tooth development by controlling the expression of TEAD1 and CTGF. Moreover, a lingually inclined conical third molar has been observed in TAZ-mutant mice (Pandya et al., 2022). However, YAP seems to play a more prominent role than TAZ in regulating enamel knot formation.

αE-catenin promotes enamel knot formation, maintains dental mesenchyme condensation and epithelial invagination, and prevents ectopic cell proliferation by inhibiting the activity of YAP and TAZ. Moreover, deletion of YAP, but not TAZ, rescues enamel knot defects caused by the loss of αE-catenin (Li et al., 2016). Similarly, in epithelial transit-amplifying (TA) cells of the mouse incisor, YAP is predominantly expressed in the nucleus, whereas TAZ is mainly expressed in the cytoplasm (Hu et al., 2017). The proliferation and differentiation of TA cells are crucial for maintaining the structural integrity of incisors and organ renewal. The induction of the ITGA3–FAK–CDC42 signaling axis promotes the expression of YAP in the nuclei of TA cells, maintains a highly proliferative state of TA cells to sustain tissue homeostasis, inhibits premature differentiation of epithelial cells, and prevents apoptosis (Li et al., 2011). Li also found that YAP was primarily expressed in the nuclei of the inner and outer enamel epithelium during the cap stage (Li et al., 2016), while TAZ was mainly expressed in the cytoplasm (Kwon et al., 2015; Niki et al., 2021). Notably, Hu found increased nuclear expression of TAZ in YAP-knockout epithelial cells (Hu et al., 2017). This is similar to an earlier study on the liver and intestine, where YAP knockout resulted in significant TAZ expression and nuclear localization, whereas YAP overexpression reduced TAZ levels (Moroishi et al., 2015). Therefore, TAZ may be activated after YAP knockdown to compensate for the loss of YAP, and there may be a negative feedback regulation of YAP and TAZ (Moroishi et al., 2015; Hu et al., 2017). These studies have also explained why YAP deficiency has a limited effect on the expression of key signaling and adhesion molecules in the epithelium.

YAP expression correlates with the timing of mouse incisor development; initially, it is present in most basal cells of the incisor epithelium, while in later stages, it is predominantly found in TA cells (Li et al., 2011; Sun Z. et al., 2018). YAP expression has also been linked to the proliferation rate. Specifically, while low YAP expression is found in slowly proliferating zones, such as the apical bud, stratum intermedium, and stellate reticulum, there is high YAP expression in the proliferating zones from the bud to eruption stages (Li et al., 2011; Li and Li, 2016). Additionally, YAP is lost in differentiated ameloblasts with the development of tooth germ (Li et al., 2011). In contrast, investigation into the development of mandibular first molars in rats showed that YAP and its homologous protein TAZ were involved in the differentiation of ameloblasts and odontoblasts, and played important regulatory roles in tooth germ development, matrix secretion and mineralization, and tooth morphogenesis (Liu et al., 2014; Zhang et al., 2017). The inconsistency may arise from differences in tooth development between mice and rats, or variances in the anatomical structure between incisors and molars. However, Liu et al. discovered that YAP and TAZ were not essential for tooth germ invagination before the bell stage of the enamel organ, indicating the presence of alternative mechanisms that compensate for their functions (Liu et al., 2015). Overall, there are both similarities and differences in the expression and localization of YAP and TAZ during tooth development, and these two molecules have distinct regulatory roles during this process.

## 4 YAP and TAZ regulate periodontal homeostasis and remodeling

**FIGURE 4:**
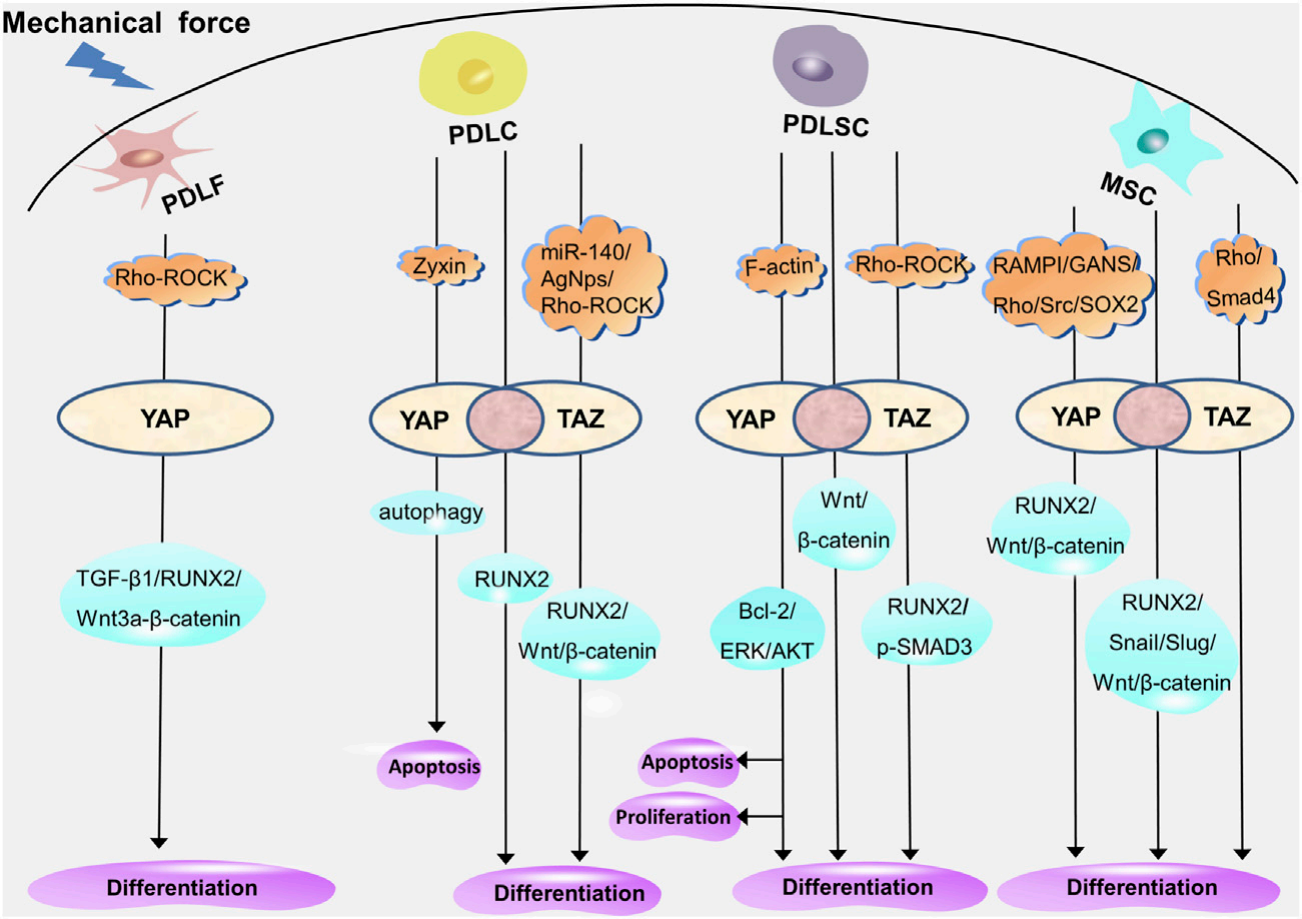
YAP and TAZ regulate the proliferation, differentiation, and apoptosis of periodontal cells through different molecular pathways. YAP mediates myofibrogenic differentiation of PDLFs through RUNX2, TGF-β1, and Wnt3a/β-catenin. In PDLCs, YAP and TAZ mediate the osteogenic differentiation through RUNX2; YAP regulates apoptosis through autophagy; and TAZ mediates the osteogenic differentiation through RUNX2, Wnt/β-catenin under the regulation of miR-140, AgNps, and Rho-ROCK. In PDLSCs, YAP and TAZ mediate osteogenic differentiation through Wnt/β-catenin; YAP mediates proliferation, differentiation, and apoptosis through ERK, Bcl-2, and AKT; and TAZ mediates the proliferation, differentiation, and apoptosis through p-SMAD3 and RUNX2 under the regulation of Rho-ROCK. In MSCs, YAP and TAZ mediate proliferation and osteogenic differentiation through RUNX2, Snail, Slug, and Wnt/β-catenin; YAP mediates osteogenic differentiation through Wnt/β-catenin and RUNX2 under the regulation of SOX2, Src, RAMPI, and GANS; and TAZ is involved in the regulation of Rho- and Smad4-mediated osteogenic differentiation. The overlap of YAP and TAZ represents the coordination of the two (pink circle), and the respective regulatory mechanisms of YAP and TAZ are shown on either side (white oval).

Recent studies have revealed that cells exhibit various biological reactions when exposed to different forces (Huang et al., 2018), including mechanical forces such as continuous or intermittent compression forces, fluid shear force, mechanical vibration, cyclic tension stress, and static tensile strain (Chen et al., 2021; Qin et al., 2021; Wu et al., 2021; Shi et al., 2022; Sun et al., 2022; Manokawinchoke et al., 2023). Mechanical force–induced OTM leads to a series of cell proliferation, differentiation, and apoptosis in periodontal tissues (Deng et al., 2021). Periodontal ligament fibroblasts (PDLFs, the main cellular component of PDLCs, also known as PDLCs (Li et al., 2021)) provide the biological basis for periodontal soft tissue remodeling through myofibroblast differentiation. Additionally, the osteogenic and osteoclastic differentiation of PDLCs, periodontal ligament stem cells (PDLSCs), and bone mesenchymal stem cells (BMSCs) located in the Periodontal ligament (PDL) and alveolar bone provide the biological basis for the reconstruction of periodontal soft tissue and bone tissue. As transcriptional coactivators, YAP and TAZ participate in the regulation of periodontal cells through different molecular pathways (Figure 4). The exploration of the mechanism of YAP and TAZ may not only help to build a healthy periodontal environment, but could also provide directions for finding potential therapeutic targets in the orthodontic process, so as to accelerate OTM and shorten the treatment time.

### 4.1 Effects of YAP and TAZ on myoblastic differentiation

The activation, proliferation, differentiation, and apoptosis of fibroblasts are essential not only for the dynamic regulation of the extracellular matrix, but also for periodontal remodeling in response to mechanical stimuli. Previous studies have demonstrated the involvement of YAP and TAZ in fibroblast activation and proliferation (Jorgenson et al., 2017; Link et al., 2022). Those findings have been confirmed in studies on periodontal fibroblasts (Table 1). For example, Kim et al. found that YAP not only influenced the morphology and cytoskeleton of PDLFs but it also regulated their proliferation and mineralization through a PDL-mimic fibrous scaffold model (Kim et al., 2023). Moreover, the application of static strain to human PDLFs (hPDLFs) results in significant nuclear translocation of YAP, which is mediated by the activation of extracellular signal regulated kinase (ERK)1/2 (Hulter-Hassler et al., 2017). Furthermore, the proliferation marker Ki-67 and nuclear YAP exhibit similar expression patterns, suggesting that the proliferation of hPDLFs is mediated by the activation of YAP in response to static strain (Huelter-Hassler et al., 2017).

**TABLE 1:** YAP regulates proliferation and differentiation of fibroblasts.

Molecule	Model	Type	Effect	Dependent signaling pathway	Study
YAP	PDL-mimic fibrous scaffolds	Surface topography	Proliferation	HIPPO-YAP	Kim et al. (2023)
YAP	HPDLFs	Static equiaxial strain	Proliferation	HIPPO-YAP	Hulter-Hassler et al. (2017)
YAP	HPDLFs	Static equiaxial strain	Proliferation	HIPPO-YAP	Huelter-Hassler et al. (2017)
YAP	HPDLCs	Cyclic uniaxial tension loading	Differentiation	RhoA/ROCK-YAP-TGF-β1/RUNX 2	He et al. (2019)
	OTM	Orthodontic loading			
YAP	HPDLFs	Tension and compression	Differentiation	Wnt3a/β-catenin	Xu et al. (2017)
	OTM			TGF-β1-JNK	
YAP	HPDLCs	Mechanical tensile stress	Differentiation	TGF-β1-HIF-1α	Watanabe et al. (2014)
	OTM	Orthodontic loading			

In recent years, some scholars have proposed that PDLFs can be transformed into myofibroblasts under mechanical stress. The process is accompanied by the upregulation of alpha-smooth muscle actin (α-SMA), which may be used as a target for regulating mechanical signal transduction and tissue remodeling in OTM. It has been confirmed that the myofibroblast marker α-SMA is upregulated in hPDLFs under cyclic mechanical tension and in a rat model of OTM (Meng et al., 2010). The expression of α-SMA mediated by the transforming growth factor-β (TGF-β)–Smad3 pathway can enhance the contractility of myofibroblasts and participate in periodontal remodeling by maintaining the tension of PDL (Wipff et al., 2007; Meng et al., 2010). Previous studies have shown that YAP and TAZ are involved in the differentiation of fibroblasts into myofibroblasts (Piersma et al., 2015; Noguchi et al., 2017; Muppala et al., 2019). This was also confirmed in the PDLFs (Table 1). Moreover, the RAS homolog gene family member A (RhoA)/Rho-associated coiled-coil kinase (ROCK) pathway is activated in a rat OTM model and in PDLCs under cyclic uniaxial tension. Upregulation of YAP and α-SMA triggers the transcription of TGF-β1 and RUNX2, which promotes myofibroblast differentiation and initiates periodontal remodeling (He et al., 2019). The inhibition of YAP significantly reduces tension-induced myofibroblast differentiation (He et al., 2019). Furthermore, exogenous TGF-β1 increases the expression of RhoA and ROCK in PDLCs; this suggests that TGF-β1 enhances the expression of YAP, providing evidence for the feedback regulation (He et al., 2019). The Wnt/β-catenin pathway, TGF-β/JNK pathway, and TGF-β/HIF-1α (hypoxia-inducible factor 1α) are also implicated in fibroblast-to-myofibroblast differentiation (Watanabe et al., 2014; Xu et al., 2017). Further studies have indicated that compared with PDLCs, myofibroblasts are more prone to expressing osteocalcin and synthesizing extracellular matrix (Xu et al., 2014). Indeed, He et al. showed that YAP in PDLCs promoted cell differentiation by activating the downstream target gene encoding RUNX2 (He et al., 2019). However, this contradicts the different expression patterns of YAP and RUNX2 in fibroblasts reported by Sun B. et al. (2018), indicating that YAP and RUNX2 may function through distinct pathways in different cells.

### 4.2 Effects of YAP and TAZ on osteogenic differentiation

Osteoblasts originate from PDLCs, PDLSCs, and BMSCs. As these are tissue-specific pluripotent cells in the periodontal ligament, their proliferation, differentiation, and apoptosis play a vital role in maintaining the balance and stability of the periodontal environment. Taken together, these processes serve as the foundation for remodeling periodontal soft and hard tissues in response to mechanical stimuli. YAP and TAZ influence the function of periodontal cells by manipulating multiple signaling pathways and cytokines (Tables 2–4).

**TABLE 2:** YAP and TAZ regulate proliferation and osteogenic differentiation of PDLCs.

Molecules	Model	Type	Effect	Dependent signaling pathway	Study
TAZ	HPDLFs	Overexpression Knockdown	Differentiation	RhoA-TAZ	Cui et al. (2021)
TAZ	HPDLFs	Knockdown	Differentiation	RhoA-TAZ	Xu et al. (2019)
YAP/TAZ	C57BL/6 mice	Gene knockout	Differentiation	YAP-RUNX2	Pandya et al. (2022)
YAP	HPDLCs	Cyclic strain	Differentiation	YAP-RUNX2	Pandya et al. (2022)
YAP	HPDLCs	Equiaxial strain	Mechanical transduction	Zyxin-YAP	Belgardt et al. (2020)
YAP	HPDLCs	Intermittent compression force	Proliferation/Differentiation	HIPPO-YAP	Klincumhom et al. (2022)
YAP	HPDLCs	Cyclic tensile strain	Proliferation/Differentiation	HIPPO-YAP	Yang et al. (2018)
YAP	HPDLCs	Low-intensity pulsed ultrasound (LIPUS)	Apoptosis	LIPUS-YAP-autophagy	Jian et al. (2023)
TAZ	Cyclic tensile strain	Cyclic tensile strain	Differentiation	Rho-ROCK-TAZ- RUNX2	Wang et al. (2020b)
				Rho-ROCK-TAZ-Wnt/β-catenin	
YAP/TAZ	OTM	Orthodontic force	Differentiation	HIPPO-YAP HIPPO-TAZ-RUNX2	Sun et al. (2018a)

**TABLE 3:** YAP and TAZ regulate proliferation and osteogenic differentiation of PDLSCs.

Molecules	Model	Type	Effect	Dependent signaling pathway	Study
YAP	PDLSCs	Knockout	Proliferation	HIPPO-YAP	Cuizhu et al. (2015)
			Apoptosis		
YAP	HPDLSCs	Knockdown	Proliferation	ERK	Wen et al. (2017)
			Apoptosis	Bcl-2	
YAP	HPDLSCs	Knockout	Proliferation	HIPPO-YAP	Dong et al. (2021)
			Differentiation/Apoptosis		
YAP	HPDLSCs	Overexpression	Proliferation	ERK	Jia et al. (2018)
			Apoptosis	Bcl-2	
YAP	HPDLSCs	Inhibition	Proliferation	ERK	Chen et al. (2019a)
			Differentiation/Apoptosis	AKT	
YAP	HPDLSCs	Overexpression/Knockdown	Differentiation	Wnt/β-catenin	Jia et al. (2019)
YAP	HPDLSCs	Cyclic stretch stress	Proliferation	LRP6-F-actin-YAP	Wang et al. (2023b)
	OTM		Differentiation		
TAZ	HPDLSC	Overexpression/Knockdown	Proliferation	TGF-β/SMAD pathway	Gu et al. (2020)
			Differentiation		
			Apoptosis		
TAZ	PDLSCs	Depletion	Proliferation	Wnt/β-catenin	Xing et al. (2019)
			Differentiation		
TAZ	OTM	Stretch force	Differentiation	HIPPO- TAZ	Wang et al. (2017)
TAZ	PDLSCs	Surface topography	Differentiation	HIPPO- TAZ	Hu et al. (2021)

**TABLE 4:** YAP and TAZ regulate proliferation and osteogenic differentiation of MSCs.

Molecules	Model	Type	Effect		Dependent signaling pathway	Study
YAP	Mice	Knockout	Proliferation Differentiation		Wnt/β-catenin	Pan et al. (2018)
YAP	BMSCs	Knockdown	Differentiation		RAMP1-YAP	Zhang et al. (2019)
YAP	BMSCs	Overexpression/Knockdown	Differentiation		αCGRP-YAP	Wang et al. (2019)
YAP	BMSCs	Surface topography	Differentiation		YAP-RUNX2	Pan et al. (2017)
TAZ	Human MSCs	Shear stress	Differentiation		Rho-TAZ	Kim et al. (2014)
TAZ	MSCs	Depletion	Differentiation		Smad4-TAZ	Park et al. (2019)
YAP/TAZ	Mice MSCs	Knockout	Differentiation		HIPPO-YAP/TAZ	Kegelman et al. (2018)
YAP/TAZ	Mice MSCs	Knockout	Differentiation		Wnt/RUNX2	Xiong et al. (2018)
YAP/TAZ	Mice	Overexpression/Knockdown	Differentiation		SOX2-YAP-Wnt/β-catenin	Seo et al. (2013)
YAP	Rats	Knockdown	Differentiation		Src/YAP-RUNX2	Zaidi et al. (2004)
YAP/TAZ	Mice MSCs	Overexpression/Knockdown	Differentiation		Snail/Slug-YAP/TAZ	Tang et al. (2016)
YAP	Mice BMSCs	Inhibit	Differentiation		GNAS-YAP	An et al. (2019)
TAZ	MSCs	ECM stiffness	Differentiation		FAK/MAPK	Hwang et al. (2015)
			Differentiation		TGF-β	
TAZ	Nanotopographic Interface	Surface topography	Differentiation		Rho GTP	Qian et al. (2017)
YAP	Human MSCs Fibrous substrates	Surface topography	Differentiation		MIF/Akt/YAP-RUNX2	Yuan et al. (2016)
YAP	Human BMSCs	Matrix stiffness	Differentiation		JNK	Barreto et al. (2017)
YAP/TAZ	Human MSCs	Surface topography	Differentiation		COL1-ROCK-F-actin-YAP/TAZ	Komatsu et al. (2018)

#### 4.2.1 YAP and TAZ regulate the proliferation and osteogenic differentiation of PDLCs

Recently, Belgardt et al. have reported that YAP is closely related to the regulation of zyxin, an intracellular mechanosensor protein located at actin polymerization sites. Zyxin acts as a transcriptional coactivator to regulate the expression of mechanosensitive genes. Moreover, the application of stretching force on PDLCs induces zyxin expression and nuclear localization, which is regulated by YAP rather than TAZ (Belgardt et al., 2020). This sheds light on how YAP interacts with the extracellular matrix in response to mechanical stimuli to mediate cytoskeletal remodeling.

The osteogenic differentiation ability of PDLCs has been demonstrated by several studies. Intermittent compression–induced YAP has been shown to mediate the proliferation and osteogenic differentiation of PDLCs and inhibit adipogenesis, whereas inhibition of YAP hinders PDLCs differentiation and promotes adipogenesis (Klincumhom et al., 2022). Moreover, Pandya showed that the application of cyclic strain and YAP/TAZ knockdown in mice blocked the osteogenic differentiation of human PDLCs (hPDLCs), while the expression of osteogenic markers (RUNX2, alkaline phosphatase, osteocalcin, and collagen I) was significantly reduced, the mineralized tissue density of alveolar bone was reduced, and osteoclast activity was increased, thereby shifting periodontal homeostasis to catabolism (Pandya et al., 2022). However, as that study solely focused on YAP knockdown and did not investigate TAZ in the cell experiment, further investigation is necessary to determine whether YAP and TAZ exhibit distinct functions *in vivo* and *in vitro*. Yang et al. observed similar results, and the increased expression of the target genes encoding CTGF and CYR61 following the application of cyclic stretch to hPDLCs confirmed the activation of YAP (Yang et al., 2018). YAP overexpression enhanced the stretch-induced osteogenic differentiation of hPDLCs, whereas YAP knockdown inhibited the process. However, the nuclear translocation of TAZ was minimal in cyclically stretched hPDLCs, suggesting limited activation of TAZ in hPDLCs and the role of YAP as the primary regulator in the osteogenic differentiation of hPDLCs (Yang et al., 2018).

However, Wang et al. provided evidence that stimulation of PDLCs by cyclic tensile stress significantly promoted the nuclear expression of TAZ and its interaction with RUNX2 (Wang Y. et al., 2020). This observation was supported by Sun et al., who revealed a positive correlation between TAZ and RUNX2 expression (Sun B. et al., 2018). Moreover, double-labeling immunofluorescence staining demonstrated colocalization of TAZ and RUNX2 in the periodontal ligament, whereas YAP and RUNX2 exhibited distinct expression patterns (Sun B. et al., 2018). This suggests that TAZ regulates periodontal remodeling through RUNX2, while YAP may employ different mechanisms to rebuild the periodontium. Inhibition of TAZ hinders the stress-induced osteogenic differentiation of PDLCs, and inhibition of the Rho/ROCK signaling pathway hinders the nuclear aggregation of TAZ and its binding to RUNX2. This results in decreased osteogenic differentiation of PDLCs, suggesting that the activation of TAZ may be mediated by the ROCK signaling pathway (Wang Y. et al., 2020). miR-140 and silver nanoparticles have been reported to inhibit and promote the expression of the downstream effector TAZ by targeting RhoA, thereby exerting different effects on the osteogenic differentiation of hPDLFs (Xu et al., 2019; Cui et al., 2021). Moreover, both *in vivo* and *in vitro* experiments have shown that stress stimuli activate the Wnt/β-catenin signaling pathway in PDLCs, resulting in a significant increase in the nuclear translocation of signature *ß*-catenin, which plays a role in bone formation (Premaraj et al., 2011; Fu et al., 2016). Furthermore, Wang et al. demonstrated that TAZ served as an upstream factor responsible for activating Wnt/β-catenin signaling during cyclic tensile stress–mediated osteogenic differentiation of PDLCs (Wang Y. et al., 2020). Jian et al. established a lipopolysaccharide (LPS)–induced periodontal ligament inflammatory environment and discovered that the mechanical signal transformed by low-intensity pulsed ultrasound activated the nuclear expression of YAP (Jian et al., 2023). This was accompanied by reduced alveolar bone resorption. In contrast, YAP knockdown aggravated PDLCs apoptosis in the inflammatory environment. It has recently been shown that Gli1^+^ cells in the periodontal ligament play a role in osteogenesis. Under tensile force, these cells are capable of sensing mechanical stimuli through YAP and consequently proliferate and differentiate into osteoblasts to regulate alveolar bone remodeling; however, YAP knockout inhibits this process (Liu et al., 2020).

YAP and TAZ actively respond to different mechanical stimuli and mediate the proliferation, differentiation, and apoptosis of PDLCs (Table 2). The OTM involves a series of remodeling events in the periodontal ligament triggered by mechanical stimuli. Hence, YAP and TAZ hold promise as potential therapeutic targets in orthodontic treatment.

#### 4.2.2 YAP and TAZ regulate the proliferation and osteogenic differentiation of PDLSCs

PDLSCs are a subset of mesenchymal stem cells (MSCs) characterized by high proliferation activity, multidirectional differentiation potential, and mechanical sensitivity (Tomokiyo et al., 2019; Yang et al., 2023). Tang et al. found that YAP silencing significantly decreased the proliferation of human PDLSCs (hPDLSCs), increased the apoptosis rate, and altered the cell cycle distribution, suggesting that YAP is involved in regulating the proliferation and apoptosis of hPDLSCs (Cuizhu et al., 2015). This finding was confirmed by subsequent studies by Wen et al. and Chen et al., who further demonstrated that the proliferation and apoptosis of hPDLSCs were closely related to ERK, Bcl-2, and AKT signaling pathways (Wen et al., 2017; Jia et al., 2018; Chen X. et al., 2019; Dong et al., 2021).

YAP is also implicated in the osteogenic differentiation of hPDLSCs. The overexpression of YAP enhances the TNF-α-induced osteogenic differentiation and mineralized nodule formation in hPDLSCs (Dong et al., 2021). Moreover, Jia suggested that YAP promoted osteogenic differentiation while inhibited adipogenic differentiation of hPDLSCs, partly through activating the Wnt/β-catenin signaling pathway via LRP6 and DVL3 (Jia et al., 2019). Additionally, upon application of cyclic tensile stress to PDLSCs, LRP6 acted as the mechanosensor to regulate mechanical stress–inducible osteogenic differentiation of PDLSCs via the F-actin/YAP cascade. LRP6 loss caused cell morphological aberration, YAP nucleoplasmic relocation, and subsequent YAP inactivation (Wang J. et al., 2023). TAZ has been reported to promote the proliferation and apoptosis of hPDLSCs (Wang et al., 2017; Xing et al., 2019; Gu et al., 2020; Hu et al., 2021). TAZ knockdown reduces hPDLSC proliferation, promotes cell apoptosis, and inhibits osteogenic differentiation (Gu et al., 2020). Furthermore, LPS-induced enhancement in TAZ activity promotes osteogenic differentiation of PDLSCs through the Wnt/β-catenin signaling pathway (Xing et al., 2019). However, TAZ knockdown reduces osteogenic differentiation of PDLSCs by suppressing CTHRC1 overexpression, thereby attenuating bone remodeling through the collaboration of osteocytes and osteoblasts (Wang et al., 2017). YAP and TAZ regulate the proliferation, differentiation, and apoptosis of PDLSCs through different mechanisms (Table 3). It remains to be verified whether these pathways intersect in YAP and TAZ.

#### 4.2.3 YAP and TAZ regulate the proliferation and osteogenic differentiation of BMSCs

Previous research has provided evidence that YAP maintains bone homeostasis by promoting osteogenesis and inhibiting adipogenesis. It has been shown that YAP can promote the differentiation and proliferation of osteoblasts, while YAP deficiency can inhibit the osteoblast differentiation of BMSCs, which may be related to the Wnt/β-catenin signaling pathway (Pan et al., 2018). Furthermore, receptor activity-modifying protein 1 (RAMP1) regulates YAP and promotes osteogenic differentiation of BMSCs induced by calcitonin gene-related peptide (Zhang et al., 2019). YAP overexpression stimulates BMSC osteogenic differentiation, while YAP inhibition induces BMSC adipogenic differentiation (Pan et al., 2017; Wang et al., 2019). Additionally, shear stress–stimulated MSCs demonstrate significant expression and nuclear localization of TAZ and enhanced MSC osteogenic differentiation. TAZ knockdown diminishes MSC osteogenic differentiation, which might be associated with the activation of the Rho signaling pathway (Kim et al., 2014). The Smad4–TAZ axis has been identified as a promoter of MSC osteogenesis independent of the TGF-β signaling, although YAP does not interact with Smad4 to regulate MSC osteogenic and adipogenic differentiation (Park et al., 2019).

The absence of YAP and TAZ in mature osteoblasts and osteocytes also leads to a reduction in the number of osteoblasts, an increase in the number of osteoclasts, and a decrease in bone mass. At the cellular level, *in vivo*, YAP/TAZ knockdown results in a dose-dependent decrease in osteoblast activity and an increase in osteoclast activity, which impairs bone proliferation and remodeling (Kegelman et al., 2018). Transcriptionally, the absence of YAP/TAZ and the inhibition of the YAP/TAZ interaction with TEAD reduces the expression of osteogenic and collagen-related genes both *in vivo* and *in vitro*. YAP and TAZ work together to regulate osteoblast activity, matrix quality, and osteoclast remodeling, thereby promoting bone development (Kegelman et al., 2018). Taken together, the results of these studies suggest a positive role of YAP and TAZ in osteoblast differentiation; however, other studies have reported opposite findings. For example, knockout of YAP and TAZ in MSCs was found to enhance osteoblast formation, possibly through activating the Wnt signaling pathway and the osteogenic factor RUNX2 (Xiong et al., 2018). Moreover, SOX2-mediated YAP overexpression inhibited MSC osteogenesis and adipogenesis by interfering with *ß*-catenin-dependent Wnt signaling (Seo et al., 2013). Moreover, the Src/YAP tyrosine kinase signal was reported to regulate bone homeostasis by inhibiting the binding of YAP to RUNX2, with YAP acting as an inhibitor of osteocalcin activation in osteoblasts (Zaidi et al., 2004). These results suggest that YAP and TAZ may play opposing roles in different stages of osteoblast differentiation and that they regulate cell differentiation through distinct signaling pathways (Table 4).

### 4.3 Effects of YAP and TAZ on osteoclastic differentiation

Osteoclast activity is considered the main rate-limiting factor for OTM. After orthodontic force, monocytes derived from hematopoietic stem cells are first recruited on the pressure side and differentiate into osteoclasts under the influence of M-CSF and RANKL. Because osteocytes contain higher levels of osteoclast markers than other cells in the periodontium, they are considered a crucial source of RANKL in alveolar bone remodeling during OTM.

Previous studies have suggested the involvement of the Hippo signaling pathway in osteoclastogenesis (Reszka et al., 1999; Lee et al., 2015; Li Q. et al., 2019). Increasing evidence suggests that YAP and TAZ regulate osteoclastogenesis and bone resorption (Yang et al., 2021). In bone marrow–derived macrophages, Zhao et al. found that YAP knockdown prevented the formation of multinucleated osteoclasts and the association of YAP with TEAD transcription factors, the protein and mRNA levels of YAP were downregulated (Zhao et al., 2018). Mechanistically, this supports the interaction between YAP/TEAD and AP-1 and cooperation with downstream gene transcription. YAP inhibition has also been found to impair RANKL-induced NF-κB signaling, thereby inhibiting osteoclastogenesis. A study on PIEZO1 has demonstrated increased bone resorption due to decreased YAP nuclear localization (Wang L. et al., 2020). In mice, knockout of YAP and TAZ results in significant increases in osteoclast markers (tartrate-resistant acid phosphatase and cathepsin K), number of osteoclasts, area of periodontal ligament hyalinization, and osteoclast activity (Pandya et al., 2022). Another study has revealed that YAP serves as an upstream regulator and induces the expression of growth differentiation factor 15 (GDF15) in PDLCs (Shiu et al., 2018). YAP upregulates the expression of proinflammatory cytokines and the ratio of RANKL/OPG to promote osteoclastogenesis. However, the inhibition of GDF15 also disrupts the nuclear translocation of NF-κB and the phosphorylation of ERK, thereby inhibiting osteoclast differentiation (Li et al., 2020).

Unfortunately, there are not enough relevant studies to explore the mechanism by which YAP and TAZ regulate osteoclastic differentiation of periodontal cells. Therefore, the effect of YAP and TAZ on osteoclast differentiation is a potential direction to explore bone resorption in OTM, which can further complement the mechanism of bone resorption under mechanical stimulation.

### 4.4 Effects of YAP and TAZ on osteocytes

Osteocytes, which originate from mature osteoblasts when they become embedded in the bone matrix, have traditionally been considered inactive bone matrix placeholder cells. Recently, they have been recognized as the major mechanosensory cells, and their importance in mechanical signal transduction is increasingly appreciated, especially in fluid shear–mediated bone remodeling (Dole et al., 2021; Liu et al., 2022). In the study by Li et al., fluid shear force activated the nuclear expression of YAP through the ion channel Piezo1 and stimulated the upregulation of Wnt1 expression in bone cells, thereby significantly increasing the bone mass and strength of mice (Li X. et al., 2019). Moreover, YAP and TAZ translocated to the nucleus and activated their target genes in a culture of bone cells, and RNA sequencing analysis revealed that YAP/TAZ knockdown mediated the regulation of several genes, including those involved in osteocyte dendrite formation (Zarka et al., 2021). These studies demonstrated the important roles of YAP and TAZ in the mechanical transduction of osteocytes and regulation of gene expression. Kegelman et al. further confirmed the important role of YAP and TAZ in osteoblast-mediated bone remodeling. YAP and TAZ control bone matrix accrual, organization, and mechanical properties by regulating osteocyte perilacunar/canalicular remodeling (Kegelman et al., 2019). Osteocyte-conditional YAP/TAZ deletion alters collagen matrix content and organization and decreases bone mass and bone mechanical properties (Kegelman et al., 2018; Kegelman et al., 2019). However, the exact mechanism by which YAP and TAZ regulate osteocyte function under mechanical stimulation needs to be further explored. This would be an interesting emerging area.

## 5 Clinical application of YAP and TAZ

In physiological conditions, YAP and TAZ serve as key regulatory factors for cell proliferation, differentiation, and apoptosis, influencing the occurrence and development of diseases, organ development, as well as tissue regeneration and repair. On one hand, excessive and uncontrolled cell proliferation may lead to the development of tumors, cancers, metabolic disorders, and fibrotic diseases. On the other hand, promoting YAP and TAZ-mediated cell proliferation holds the potential for organ regeneration in aging and damaged organs and tissues. Therefore, YAP and TAZ have been extensively studied as potential targets for disease treatment and tissue regeneration (Yoo et al., 2021; Piccolo et al., 2022; Wang H. et al., 2023). Numerous studies have demonstrated the integration of YAP and TAZ with various biomaterials and biotechnologies in dental tissue remodeling and engineering. These include controlling basal stiffness and topography, designing cell shape and alignment through micropatterning, and cellular mechanical stretching, among others (Yang et al., 2023). Currently, most research on YAP and TAZ focuses on indirectly targeting their activation by molecules such as blebbistatin, Cytochalasin D, Y-27, XAV939, and C3 toxin (Yang et al., 2018; He et al., 2019; Xu et al., 2019; Wang Y. et al., 2020; Wei et al., 2020; Ehlinger et al., 2021; Hu et al., 2021). Verteporfin acts as a direct inhibitor of YAP (Belgardt et al., 2020; Li et al., 2020; Lee et al., 2022). However, the majority of these interventions are still at the cellular or animal experimental stages. Verteporfin, Super-TDU and CA3 are currently recognized drugs that inhibit YAP by blocking the interaction between YAP/TAZ and TEAD, reducing YAP1 expression, and attenuating YAP1 transcriptional activity (Jiao et al., 2014; Song et al., 2018; Barrette et al., 2022). CK666, trichostatin A and agrin have been reported as chemical activators of YAP/TAZ by promoting nuclear accumulation and inducing YAP release (Bassat et al., 2017; Ehlinger et al., 2021). Although these inhibitors and activators are considered to play important roles in clinical diseases, further clinical trials are needed to validate their pharmacological effects in oral diseases. Moreover, their safety for application in humans requires additional clinical research.

In orthodontic treatment, proper tissue remodeling and accelerated tooth movement are desirable outcomes. Previous studies have indicated various methods for accelerating tooth movement, such as local cytokine injections, minimally invasive trauma, low-level laser therapy, and vibration as non-invasive approaches (Li et al., 2021). However, the targeted treatment of YAP and TAZ for accelerating orthodontic tooth movement is still in its early exploration stage. Considering the established biological functions of YAP and TAZ and the complexity of orthodontic tooth movement, it is urgent to explore novel strategies targeting YAP and TAZ to accelerate tooth movement and reveal potential therapeutic applications.

## 6 Conclusion and perspective

YAP and TAZ are key transcription factors in the Hippo pathway and play a vital role in regulating cell proliferation, differentiation, and apoptosis. Their mutation or dysregulation results in various developmental and regenerative defects in tissues and organs, ultimately leading to significant consequences. In recent years, extensive studies have been conducted on YAP and TAZ. These two molecules are of significant interest to dentists owing to their ability to regulate normal tooth development, provide a balanced and stable periodontal environment, and mediate periodontal remodeling. The regulation of the periodontium and tooth development by YAP and TAZ is complex and encompasses multiple aspects. Although some studies have shed light on their functions, a full list of functions and specific mechanisms require further investigation. As follows:

1. YAP and TAZ become crucial regulators for cell proliferation, differentiation, and apoptosis in different physiological environments, responding to mechanical cues. Although several possible molecular pathways have been discussed, there are still unidentified mechanisms and related factors that require further exploration.
2. As mentioned previously, the diversity of mechanical cues in the body presents different parameters in terms of tensile stress, compressive stress, and fluid shear stress, with variations in frequency, quantity, or duration. Therefore, it is necessary to further elucidate, from the perspective of mechanobiology, whether different mechanical forces lead to differential expression of YAP and TAZ in the nucleus or cytoplasm, as well as whether the differences in cellular function are related to mechanical forces.
3. YAP and TAZ exhibit synergistic and distinct effects on tissues due to their inherent differences. However, most previous studies did not distinguish between YAP and TAZ, overlooking their discrepancies in these processes and the underlying causes. Therefore, it is crucial to differentiate between YAP and TAZ to gain a comprehensive understanding of their respective roles.
